# Glucagon-like peptide-1 receptor agonists and tirzepatide are associated with reduced mortality, cardiovascular, and psychiatric risks in patients with hidradenitis suppurativa and type 2 diabetes and/or obesity: a retrospective cohort study

**DOI:** 10.3389/fmed.2026.1909537

**Published:** 2026-08-31

**Authors:** Inga C. Brouer, Aida Zani, Henning Olbrich, Ralf J. Ludwig, Diamant Thaçi

**Affiliations:** 1 Institute for Inflammation Medicine, University of Lübeck, Lübeck, Germany; 2 Lübeck Institute of Experimental Dermatology, University of Lübeck and University Clinic of Schleswig-Holstein, Lübeck, Germany

**Keywords:** diabetes mellitus, GLP-1 receptor agonist, hidradenitis suppurativa, incretin-based therapy, obesity, real-world, tirzepatide, TriNetX

## Abstract

**Background:**

Hidradenitis suppurativa (HS) is a chronic inflammatory skin disease strongly associated with obesity and diabetes mellitus type 2 (T2D). GLP-1 receptor agonists (GLP-1RAs) and tirzepatide are increasingly used for metabolic control, but their systemic effects in HS remain poorly characterized.

**Objectives:**

To compare all-cause mortality, cardiovascular, rheumatologic, metabolic, psychiatric, and non-communicable inflammatory outcomes in HS patients with T2D or obesity treated with GLP-1RAs/tirzepatide vs. alternative metabolic medications.

**Methods:**

This retrospective cohort study used the US Collaborative Network in TriNetX. Adults (≥18 years) with HS and T2D or obesity were included, excluding prior bariatric surgery. Exposed patients received a GLP-1RA or tirzepatide for at least one year, controls received other systemic antidiabetic or anti-obesity agents. Outcomes over a 2-year follow-up were assessed using 1:1 propensity score matching (PSM) for each outcome category. Outcomes included all-cause mortality, cardiovascular, rheumatologic, metabolic, psychiatric, and non-communicable inflammatory events. Hazard ratios (HRs) with 95% confidence intervals (CIs) were calculated, and Cox regression was used to confirm significant findings. Sensitivity analyses and benchmark comparisons were performed.

**Results:**

Before matching, 8,564 patients received GLP-1RA/tirzepatide and 6,299 alternative therapies. After matching, GLP-1RA/tirzepatide use was associated with reduced all-cause mortality (HR: 0.24, 95% CI: 0.145–0.395; *p* < 0.0001) and major adverse cardiovascular events (MACE) (HR: 0.61, 95% CI: 0.500–0.749; *p* < 0.0001). Risks of stroke and heart failure were also reduced (both *p* < 0.05). Psychiatric outcomes, including depression (HR: 0.78), suicidal ideation (HR: 0.38), and substance use disorder (HR: 0.58), were significantly reduced (all *p* < 0.05). Risks for rheumatologic and non-communicable chronic inflammatory outcomes were similar among groups. A modest increase in the risk of hyperlipidemia (HR: 1.25), metabolic dysfunction-associated steatotic liver disease (MASLD) (HR: 1.37), and nausea/vomiting (HR: 1.31) was observed in the GLP-1RA/tirzepatide group compared with alternative therapies (all *p* < 0.05).

**Conclusion:**

GLP-1RAs and tirzepatide are associated with reduced mortality, cardiovascular and psychiatric morbidity in HS patients with obesity or T2D, without increasing rheumatologic or inflammatory risk. These results extend the systemic benefits of GLP-1RAs and tirzepatide beyond metabolic control and suggest potential therapeutic value in HS.

## 1 Introduction

GLP-1 receptor agonists (GLP-1RAs) and dual GLP-1/GIP receptor agonists including tirzepatide are increasingly used in clinical practice for obesity and diabetes mellitus type 2 (T2D) (1). Given the high prevalence of obesity and T2D among patients with hidradenitis suppurativa (HS), these individuals are frequently exposed to incretin-based treatments (2). While the impact on the metabolic comorbidity is known, the potential impact on inflammation and disease-associated comorbidities in HS remains to be clarified (3).

GLP-1RAs mimic endogenous GLP-1, an incretin hormone secreted by intestinal L-cells. They act via GLP-1 receptors in the hypothalamic satiety center, reducing food intake, slowing gastric emptying, and modulating vascular, muscle, and adipose tissue function (4). Dual GLP-1/GIP agonists, such as tirzepatide, additionally activate GIP receptors, providing superior weight loss and glycemic control (5, 6).

HS is a chronic inflammatory skin disease with an estimated global prevalence of approximately 2.5% (7). HS is strongly linked to obesity, T2D, and multiple systemic comorbidities including cardiovascular disease and psychiatric disorders, which contribute to patient morbidity and mortality (8).

Small case series and retrospective studies suggest that GLP-1RAs and GLP-1/GIP agonists may reduce HS flares (9–11). Retrospective data support the potential benefit of GLP-1RAs in HS: a retrospective cohort study reported that GLP-1RA use in HS patients was associated with fewer surgical interventions and hospitalizations (12). The study compared HS patients receiving GLP-1RAs to HS patients without GLP-1RA exposure, matched to *n* = 11,950 per group with matching variables not fully reported. GLP-1RA users had significantly lower rates of emergency visits, antibiotic prescriptions, pain medications, incision and drainage procedures, and lower mortality (all RR <1.0) over 10-year follow-up. Another real-world cohort study examined GLP-1RAs effect specifically on cardiovascular risk reduction in HS patients (13). It compared GLP-1RA users to non-users (*n* = 19,920) using 1:1 propensity score matching (PSM) on age, sex, race, ethnicity, and major adverse cardiovascular events (MACE), showing significantly lower odds of MACE components. Additionally, an open-label, single-center proof-of-concept study evaluated tirzepatide in 20 adults with moderate-to-severe HS [Physician's Global Assessment (PGA) ≥3; Body mass index (BMI) ≥27] over 24 weeks. At week 24, 80% achieved HS Clinical Response (HiSCR), with improvements in PGA, Dermatology Life Quality Index (DLQI), Visual Analog Scale (VAS), and Hospital Anxiety and Depression Scale (HADS). The treatment was well-tolerated with favorable metabolic effects (14).

However, current evidence relies on small samples, short follow-up, and lacks active metabolic comparators. No large real-world analysis has yet compared GLP-1RAs or tirzepatide vs. alternative antidiabetic or anti-obesity drugs in HS patients for broad systemic outcomes, including all-cause mortality. This study addresses these gaps by conducting a retrospective cohort study using the TriNetX US Collaborative Network. Four parallel analyses were performed to assess the impact of incretin-based therapy on HS patients, benchmark populations, and HS-specific treatment effects. The investigation focuses on systemic outcomes, including mortality, rheumatologic-, cardiovascular-, metabolic-, psychiatric-, and inflammatory events. By providing comprehensive real-world evidence beyond metabolic effects alone, this study aims to inform holistic management strategies for HS patients exposed to incretin-based therapies.

## 2 Methods

### 2.1 Study design and database

This retrospective cohort study utilized the US Collaborative Network within the TriNetX research platform, following established protocols (15). To assess the specific impact of GLP-1RA and tirzepatide in patients with HS, we defined a cohort of adults (≥18 years) with HS and comorbid T2D or obesity, comparing GLP-1RA/tirzepatide users with patients receiving no GLP-1RA/tirzepatide but alternative antidiabetic or anti-obesity medications.

In parallel, three additional benchmark analyses were performed: [1] adults with T2D or obesity without HS, comparing GLP-1RA/tirzepatide vs. alternative therapies; [2] adults with T2D or obesity not exposed to GLP-1RA/tirzepatide, comparing patients with HS vs. no HS; [3] adults with T2D or obesity receiving GLP-1RA/tirzepatide, comparing patients with HS vs. no HS.

**Figure 1:**
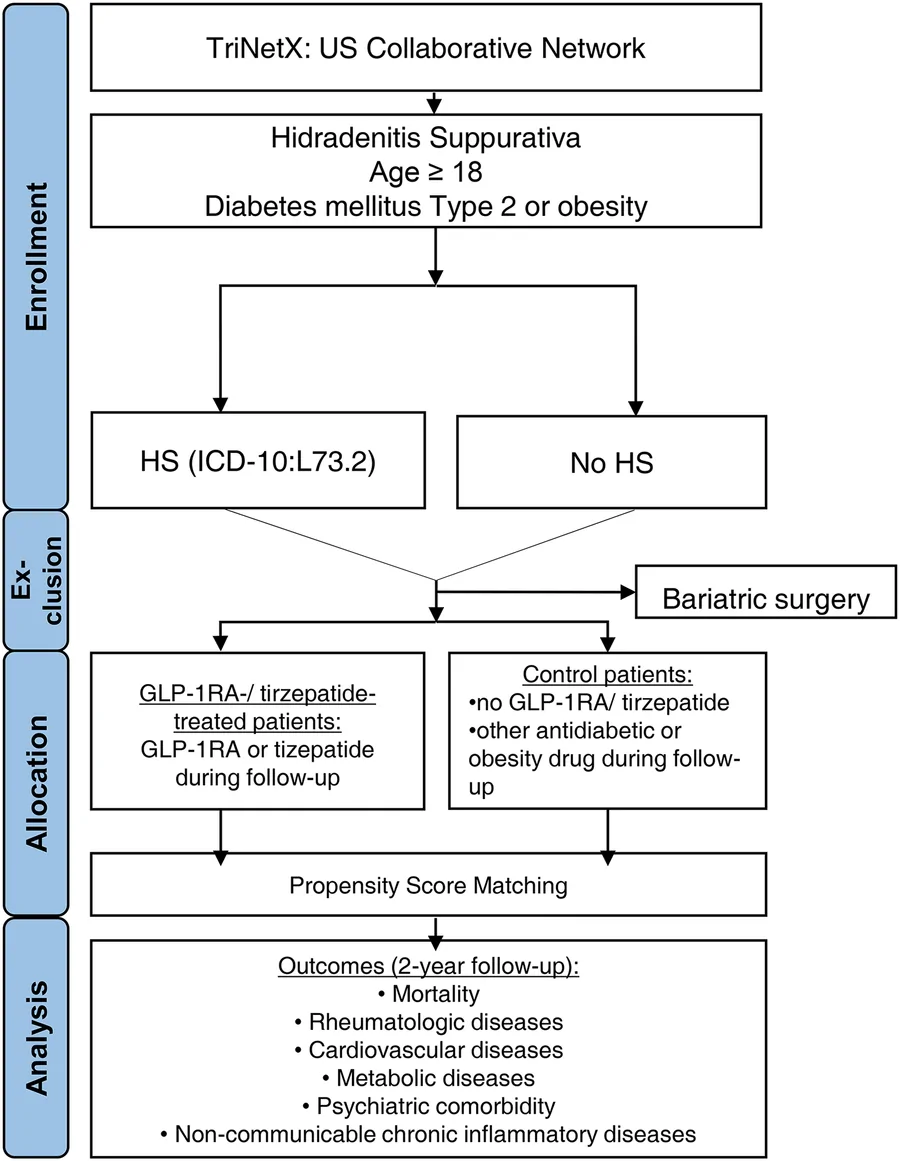
Flowchart of study inclusion. GLP-1RA, glucagon-like peptide-1 receptor agonist; ICD-10, International Classification of Diseases, Tenth Revision.

The index event was defined as initiation of GLP-1RA or tirzepatide in the exposed group, or initiation of alternative antidiabetic/anti-obesity therapy in controls. Patients with prior bariatric surgery were excluded. Outcomes were assessed over a 2-year follow-up period. Figure 1 illustrates the study design.

### 2.2 Study population

Data were retrieved in February 2026 from the TriNetX US Collaborative Network. We identified adults aged ≥18 years with HS (ICD-10: L73.2) requiring systemic therapy (secukinumab (RxNorm 1599788), adalimumab (RxNorm 327361), bimekizumab (RxNorm 2668041), clindamycin (RxNorm 2582), or abscess incision (CPT 1007164)) and comorbid T2D (E08–E13) or obesity (E65–E68). Patients with prior bariatric surgery (ICD-10: Z98.84; CPT 1007385) were excluded. Cases (*n* = 8,564) included patients treated with GLP-1RAs or tirzepatide [including all GLP1-RAs listed in ATC-chapter A10BJ + tirzepatide (RxNorm 2601723)] for ≥1 year. Controls (*n* = 6,299) comprised patients not treated with GLP-1RAs or tirzepatide but receiving alternative antidiabetic/anti-obesity agents: orlistat (RxNorm 37925), metformin (RxNorm 6809), alpha glucosidase inhibitors (ATC A10BF), sulfonylureas (ATC A10BB), dipeptidyl peptidase 4 inhibitors (ATC A10BH), sodium-glucose co-transporter 2 inhibitors (ATC A10BK), thiazolidinediones (ATC A10BG), phentermine (RxNorm 8152) with topiramate (RxNorm 38404), naltrexone (RxNorm 7243) with bupropion (RxNorm 42347)) for comparable duration. The study size was determined by the number of eligible patients available in the TriNetX US Collaborative Network meeting the predefined inclusion and exclusion criteria during the study period.

Additional cohorts were generated for the three benchmark analyses.

### 2.3 Outcomes

All-cause mortality was assessed over the 2-year follow-up period. Additional outcomes included rheumatologic, cardiovascular, metabolic, psychiatric, and substance use disorders (SUD), as well as non-communicable chronic inflammatory diseases (NCID) (Supplementary Table 1). A negative control outcome (presbyopia) was included to evaluate potential residual bias. Each outcome category underwent separate 1:1 PSM analyses.

### 2.4 Analyses and statistics

Analyses were performed using the TriNetX platform. Patients were stratified according to treatment exposure (GLP-1RA/Tirzepatide vs. alternative metabolic agents) and disease status (HS vs. non-HS) for the four predefined analyses. PSM was conducted separately for each outcome category, controlling for demographics, comorbidities, and concomitant medications. Matching was performed 1:1 using a greedy nearest-neighbor algorithm with a caliper distance of 0.1 pooled standard deviations. Covariate balance before and after matching was evaluated using standardized mean differences (SMDs), with values below 0.1 considered indicative of adequate balance. After matching, all covariates achieved SMDs below 0.1, confirming satisfactory balance between groups. Measures of association included risk difference, risk ratio, odds ratio, and hazard ratio (HR) with 95% confidence interval (CI). Kaplan–Meier analysis and log rank testing assessed time to event differences. HRs were derived from Cox proportional hazards models. Patients with preexisting outcomes were excluded. The Holm Šidák correction was applied within each outcome category to adjust for multiple comparisons. The significance level *α* was set at 0.05.

For model triangulation, multivariable Cox proportional hazards models were performed for significant endpoints from the primary analysis. These models included the same covariates used in PSM for the respective outcome, as well as healthcare utilization (visit frequency prior to index) to account for potential detection bias. Covariates comprised age at index, sex, race/ethnicity, cardiovascular risk factors, comorbidities, and concomitant medications, all assessed within one day prior to the index date. HR with 95% CI were reported. Outcomes were assessed over a 2-year follow-up period after the index date.

To assess the robustness of the results in the main analysis, five predefined sensitivity analyses were performed. S1 reran the analysis two weeks later to check result stability. In S2, all events occurring within the first three months after the index date were excluded to mitigate protopathic bias. S3 repeated the primary comparison in a subgroup restricted to HS patients with obesity but without T2D, to evaluate whether cardiovascular associations were independent of diabetes-related effects. PSM was repeated within this subgroup using the same covariates as the primary analysis. For S4 the primary comparison was replicated after excluding all patients receiving adalimumab, to assess whether biologic therapy, used as an inclusion criterion for HS-directed systemic treatment, confounded the observed cardiovascular associations. S5 addressed potential immortal time bias arising from the ≥1-year exposure requirement for cohort inclusion. The risk period was redefined to begin 1 year after the index date and extend to 2 years, using the same PSM cohorts as the primary analysis. Patients with the outcome prior to the landmark were excluded from the risk set.

### 2.5 Ethics statement

All data retrieved from TriNetX were aggregated and de-identified. Institutional Review Board approval and written informed consent were not required. Data handling complies with HIPAA §164.514(a) standards.

## 3 Results

### 3.1 Study population characteristics

A total of 14,863 adults with HS and T2D or obesity were included in the primary analysis. Among them, 8,564 received GLP-1RAs or tirzepatide, and 6,299 received other antidiabetic or anti-obesity medications without GLP-1RA/tirzepatide exposure. Prior to matching, the GLP-1RA/tirzepatide group had a significantly higher proportion of females, and a greater prevalence of obesity, hypertension, T2D, and dyslipidemia, with SMDs exceeding 0.20. After PSM all baseline covariates were well balanced, with all SMDs below 0.025. Baseline characteristics before and after PSM of both cohorts are presented in Table 1.

**Table 1:** Baseline characteristics of patients with hidradenitis suppurativa (HS) treated with glucagon-like peptide-1 receptor agonist (GLP-1RA) or tirzepatide compared with patients treated with other antidiabetic or obesity medications, before and after propensity-score matching (PSM). PSM was performed separately for each outcome category (all-cause mortality, cardiovascular, rheumatological, metabolic, psychiatric, and non-communicable inflammatory outcomes). The characteristics shown refer to the matched cohort for all-cause mortality (*n* = 5,234 per group).

			Before matching				After matching			
Category	Characteristic	Code	GLP 1/Tirzepatide (*n* = 8,564)	Other drug (*n* = 6,299)	*p*-value	SMD	GLP 1/Tirzepatide (*n* = 5,234)	Other drug (*n* = 5,234)	*p*-value	SMD
	Age at Index (years), mean ± SD	—	44.5 ± 12.6	44.4 ± 14.8	0.6086	0.0084	43.9 ± 12.6	43.8 ± 14.9	0.9532	0.0011
Sex	Female	F	7,071 (82.567%)	4,733 (75.139%)	<0.0001	0.1826	4,157 (79.423%)	4,164 (79.557%)	0.8654	0.0033
	Male	M	1,485 (17.34%)	1,563 (24.813%)	<0.0001	0.1840	1,075 (20.539%)	1,067 (20.386%)	0.8463	0.0038
Race/ethnicity	White	2106-3	4,469 (52.184%)	3,121 (49.548%)	0.0015	0.0527	2,670 (51.013%)	2,652 (50.669%)	0.7249	0.0069
	Hispanic or Latino	2135-2	696 (8.127%)	608 (9.652%)	0.0012	0.0536	467 (8.922%)	485 (9.266%)	0.5406	0.0120
	Asian	2028-9	174 (2.032%)	131 (2.08%)	0.8386	0.0034	110 (2.102%)	107 (2.044%)	0.8369	0.0040
Diagnoses	Overweight and obesity	ICD-10-CM E66	7,286 (85.077%)	4,126 (65.502%)	<0.0001	0.4660	4,028 (76.958%)	3,995 (76.328%)	0.4459	0.0149
	Essential (primary) hypertension	ICD-10-CM I10	5,615 (65.565%)	3,421 (54.31%)	<0.0001	0.2312	2,962 (56.592%)	2,983 (56.993%)	0.6786	0.0081
	Diabetes mellitus	ICD-10-CM E08–E13	5,696 (66.511%)	3,400 (53.977%)	<0.0001	0.2582	2,845 (54.356%)	2,873 (54.891%)	0.5825	0.0107
	Disorders of lipoprotein metabolism & other lipidemias	ICD-10-CM E78	5,208 (60.813%)	2,930 (46.515%)	<0.0001	0.2897	2,600 (49.675%)	2,611 (49.885%)	0.8297	0.0042
	Chronic lower respiratory diseases	ICD-10-CM J40-J4A	3,830 (44.722%)	2,361 (37.482%)	<0.0001	0.1475	2,047 (39.11%)	2,090 (39.931%)	0.3900	0.0168
	Nicotine dependence	ICD-10-CM F17	3,082 (35.988%)	2,396 (38.038%)	0.0105	0.0425	1,917 (36.626%)	1,952 (37.295%)	0.4785	0.0139
	Ischemic heart diseases	ICD-10-CM I20-I25	1,369 (15.986%)	848 (13.462%)	<0.0001	0.0712	708 (13.527%)	708 (13.527%)	1.0000	<0.0001
	Socioeconomic/psychosocial hazards	ICD-10-CM Z55–Z65	1,013 (11.829%)	567 (9.001%)	<0.0001	0.0927	501 (9.572%)	512 (9.782%)	0.7161	0.0071
	Chronic kidney disease (CKD)	ICD-10-CM N18	900 (10.509%)	449 (7.128%)	<0.0001	0.1194	375 (7.165%)	403 (7.7%)	0.2968	0.0204
	Alcohol related disorders	ICD-10-CM F10	489 (5.71%)	464 (7.366%)	<0.0001	0.0670	339 (6.477%)	341 (6.515%)	0.9368	0.0016
	Cerebral infarction	ICD-10-CM I63	347 (4.052%)	200 (3.175%)	0.0050	0.0470	176 (3.363%)	164 (3.133%)	0.5082	0.0129
	Malignant neoplasm of breast	ICD-10-CM C50	178 (2.078%)	103 (1.635%)	0.0499	0.0328	78 (1.49%)	92 (1.758%)	0.2790	0.0212
	Malignant neoplasm of bronchus and lung	ICD-10-CM C34	34 (0.397%)	31 (0.492%)	0.3851	0.0143	25 (0.478%)	25 (0.478%)	1.0000	<0.0001
	Malignant melanoma of skin	ICD-10-CM C43	29 (0.339%)	29 (0.46%)	0.2394	0.0193	24 (0.459%)	25 (0.478%)	0.8861	0.0028
	Malignant neoplasm of prostate	ICD-10-CM C61	22 (0.257%)	30 (0.476%)	0.0252	0.0363	15 (0.287%)	14 (0.267%)	0.8525	0.0036
	Malignant neoplasm of colon	ICD-10-CM C18	33 (0.385%)	21 (0.333%)	0.6030	0.0087	14 (0.267%)	15 (0.287%)	0.8525	0.0036
	Malignant neoplasm of rectosigmoid junction	ICD-10-CM C19	10 (0.117%)	10 (0.159%)	0.4902	0.0113	10 (0.191%)	10 (0.191%)	1.0000	<0.0001
	Malignant neoplasm of rectum	ICD-10-CM C20	10 (0.117%)	10 (0.159%)	0.4902	0.0113	10 (0.191%)	10 (0.191%)	1.0000	<0.0001
Medications	Cardiac therapy	ATC C01	7,012 (81.878%)	4,537 (72.027%)	<0.0001	0.2355	3,967 (75.793%)	3,979 (76.022%)	0.7839	0.0054
	Insulin	VA HS501	3,586 (41.873%)	1,688 (26.798%)	<0.0001	0.3216	1,500 (28.659%)	1,533 (29.289%)	0.4771	0.0139

HS, hidradenitis suppurativa; SMD, standardized mean difference; GLP-1RA, glucagon-like peptide-1 receptor agonist; PSM, propensity-score matching.

### 3.2 Decreased cardiovascular and psychiatric risk in HS patients treated with GLP-1RA/tirzepatide

GLP-1RA/tirzepatide treatment was associated with lower all-cause mortality (HR: 0.240, 95% CI 0.145–0.395, *p* < 0.0001).

Cardiovascular outcomes showed significantly lower associated risk in the treatment group, including MACE (HR: 0.612, 95% CI: 0.500–0.749, *p* < 0.0001), myocardial infarction (HR: 0.661, 95% CI: 0.489–0.894, *p* = 0.0068), stroke (HR: 0.631, 95% CI: 0.451–0.884, *p* = 0.0069), heart failure (HR: 0.505, 95% CI: 0.395–0.645, *p* < 0.0001), ischemic heart disease (HR: 0.748, 95% CI: 0.606–0.922, *p* = 0.0063), and conduction disorders (HR: 0.725, 95% CI: 0.556–0.945, *p* = 0.0170).

GLP-1RA/tirzepatide use was also associated with a reduced risk of psychiatric outcomes, including suicidal ideation (HR: 0.379, 95% CI: 0.249–0.578, *p* < 0.0001), substance use disorder (HR: 0.578, 95% CI: 0.455–0.733, *p* < 0.0001), and depression (HR: 0.779, 95% CI: 0.670–0.906, *p* = 0.0012).

Results remained consistent in both sensitivity analyses S1 and S2. S1, which replicated the analysis at a later data extraction time point to verify the reproducibility and temporal stability of the findings (Supplementary Table 4), and S2, which excluded events within the first three months post-index to address protopathic bias (Supplementary Table 5). To evaluate whether the cardiovascular associations were independent of T2D status, a sensitivity analysis (S3) was performed in HS patients with obesity but without T2D (*n* = 1,875 per group after matching; Supplementary Table 6). In this subgroup, the composite MACE endpoint showed no association (HR: 1.006, 95% CI: 0.577–1.751). HR for heart failure (HR: 0.879, 95% CI: 0.429–1.802), ischemic heart disease (HR: 0.706, 95% CI: 0.404–1.233), and conduction disorders (HR: 0.843, 95% CI: 0.485–1.463) remained directionally consistent with the primary analysis but did not reach statistical significance. Myocardial infarction, stroke, and all-cause mortality could not be estimated due to insufficient event counts in this smaller, lower-risk subgroup. S4 excluded all adalimumab users (Supplementary Table 7). HR remained consistent with the primary analysis and statistically significant for all cardiovascular outcomes. A landmark sensitivity analysis (S5) restricting the risk period to 1–2 years post-index, designed to address potential immortal time bias from the ≥1-year exposure requirement, confirmed the primary associations for MACE (HR: 0.633, 95% CI: 0.490–0.817, *p* = 0.0004), stroke (HR: 0.512, 95% CI: 0.332–0.787, *p* = 0.0019), heart failure (HR: 0.541, 95% CI: 0.396–0.740, *p* < 0.0001), and all-cause mortality (HR: 0.399, 95% CI: 0.238–0.669, *p* = 0.0003). The HRs for myocardial infarction, ischemic heart disease, and conduction disorders were comparable to those observed in the primary analysis, with substantially overlapping CI, but did not reach statistical significance (Supplementary Table 8).

Additional Cox models further supported the association of GLP-1RA/tirzepatide use with lower risk of mortality, cardiovascular, and psychiatric outcomes (Supplementary Figure 1).

**Figure 2:**
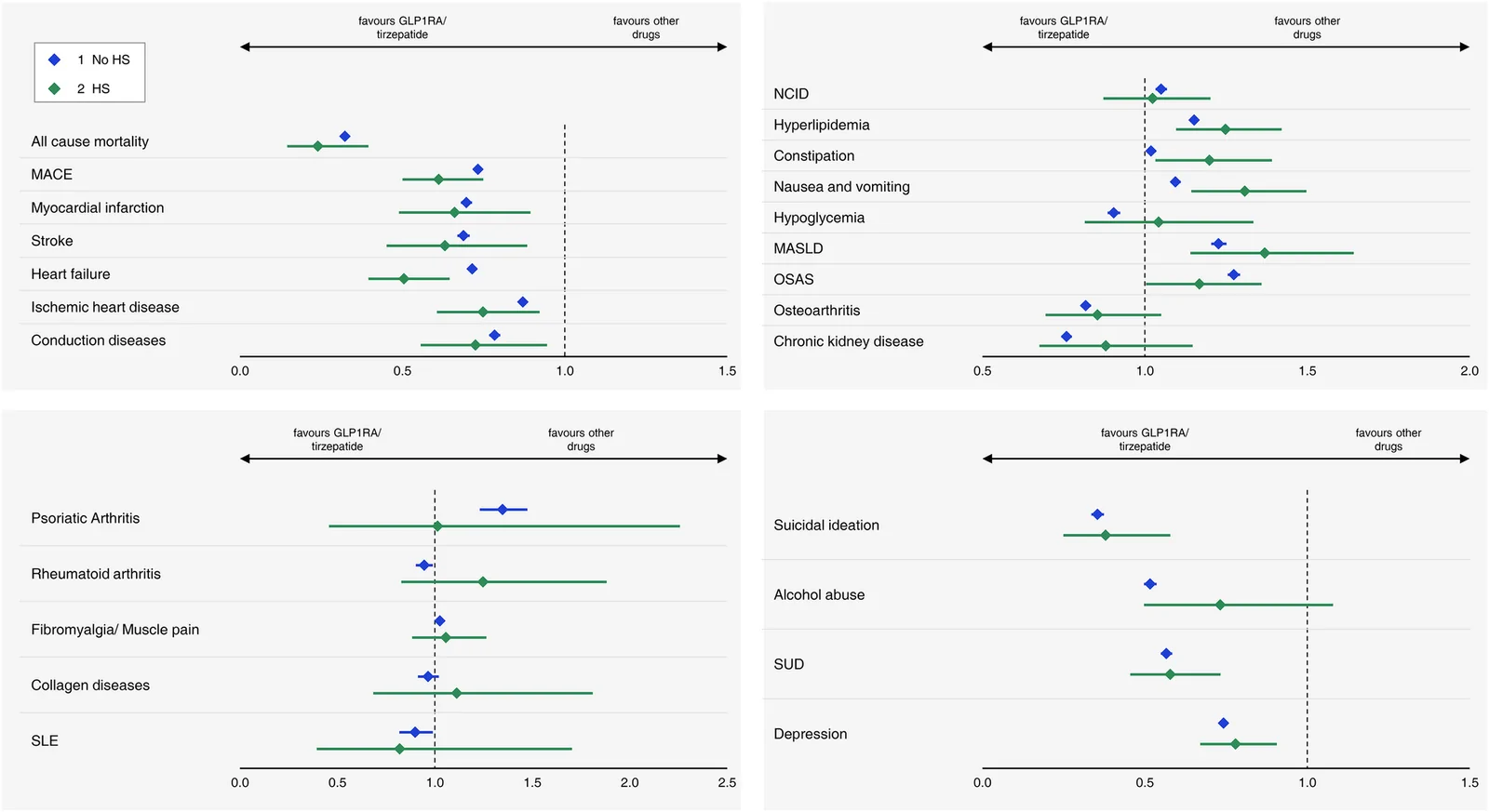
Forest plots of hazard ratios (HRs) for outcomes associated with glucagon-like peptide-1 receptor agonist (GLP-1RA) or tirzepatide treatment compared with alternative metabolic therapies in patients with and without hidradenitis suppurativa (HS). **(A)** All-cause mortality and cardiovascular outcomes; **(B)** inflammatory and metabolic outcomes; **(C)** rheumatological outcomes; and **(D)** psychiatric outcomes. HRs and 95% confidence intervals (CIs) are shown. Group 1 represents patients without HS and group 2 represents patients with HS. Outcomes were assessed from 1 day to 2 years after treatment initiation. HS, hidradenitis suppurativa; GLP-1RA, glucagon-like peptide-1 receptor agonist; HR, hazard ratio; CI, confidence interval; MACE, major adverse cardiovascular events; NCID, non-communicable chronic inflammatory diseases; MASLD, metabolic dysfunction-associated steatotic liver disease; OSAS, obstructive sleep apnoea syndrome; SLE, systemic lupus erythematosus; SUD, substance use disorder.

To assess whether these associations were specific to HS or reflected broader treatment effects, the same comparison was performed in adults with T2D or obesity without HS. In this non-HS cohort, GLP-1RA/tirzepatide treatment was likewise associated with lower all-cause mortality, cardiovascular outcomes, and psychiatric outcomes, including alcohol abuse (Figure 2, Table 2). Effect sizes for all-cause mortality and cardiovascular outcomes were larger in the HS cohort than in the non-HS cohort. To explore whether this reflected a higher baseline cardiovascular risk associated with HS itself, we compared HS and non-HS patients within each treatment stratum. Among patients not receiving GLP-1RA/tirzepatide, HS was associated with a higher MACE risk (HR: 1.184, 95% CI: 1.009–1.389, *p* = 0.0383; Supplementary Table 2), which did not remain significant after correction for multiple comparisons. No other cardiovascular outcome differed significantly. Among patients receiving GLP-1RA/tirzepatide no significant differences were observed for any cardiovascular outcome.

**Table 2:** Absolute event counts for each outcome, hazard ratios (HRs) with 95% confidence intervals (CIs), and *p*-values for outcomes associated with GLP-1 receptor agonists or tirzepatide compared with alternative metabolic therapies in patients with obesity or type 2 diabetes (T2D), stratified by hidradenitis suppurativa (HS) status (HS vs. no HS).

		Patients with obesity or T2D and HS treated with GLP-1RA compared to comparator treatment			Patients with obesity or T2D and not diagnosed with HS, treated with GLP-1RA compared to comparator treatment		
Category	Outcome	Events (treatment/control)	HR (95% CI)	*P*-value	Events (treatment/control)	HR (95% CI)	*P*-value
All-cause mortality	All-cause mortality	19/81	0.240 (0.145, 0.395)	<0.0001**	3,303/10,391	0.323 (0.310, 0.336)	<0.0001**
Rheumatologic outcomes	Psoriatic Arthritis	12/12	1.014 (0.456, 2.257)	0.9728	1,098/818	1.347 (1.230, 1.474)	<0.0001**
	Rheumatoid Arthritis with RF	51/41	1.247 (0.827, 1.881)	0.2919	3,554/3,761	0.945 (0.902, 0.989)	0.0153**
	Fibromyalgia/Muscle Pain	244/236	1.057 (0.883, 1.264)	0.5469	18,612/18,346	1.026 (1.006, 1.047)	0.0126**
	Collagen Diseases/Mixed CTD	34/31	1.112 (0.684, 1.810)	0.6677	2,495/2,591	0.965 (0.913, 1.020)	0.2057
	SLE	13/16	0.819 (0.394, 1.703)	0.5920	803/894	0.899 (0.817, 0.989)	0.0280
Cardiovascular outcomes	MACE	154/246	0.612 (0.500, 0.749)	<0.0001**	23,661/30,572	0.733 (0.721, 0.746)	<0.0001**
	Myocardial infarction	70/107	0.661 (0.489, 0.894)	0.0068**	11,137/15,727	0.697 (0.680, 0.714)	<0.0001**
	Stroke	55/88	0.631 (0.451, 0.884)	0.0069**	8,607/12,344	0.688 (0.669, 0.707)	<0.0001**
	Heart failure	97/189	0.505 (0.395, 0.645)	<0.0001**	17,036/22,888	0.715 (0.701, 0.730)	<0.0001**
	Ischemic heart disease	153/204	0.748 (0.606, 0.922)	0.0063**	27,544/31,567	0.871 (0.857, 0.885)	<0.0001**
	Conduction diseases	94/131	0.725 (0.556, 0.945)	0.0170**	14,980/18,919	0.784 (0.767, 0.801)	<0.0001**
Metabolic diseases	Hyperlipidemia	499/423	1.248 (1.096, 1.421)	0.0008 **	61,308/60,249	1.152 (1.139, 1.165)	<0.0001**
	Constipation	375/320	1.199 (1.032, 1.391)	0.0172	34,407/34,098	1.019 (1.004, 1.034)	0.0145
	Nausea and vomiting	463/391	1.308 (1.143, 1.497)	<0.0001**	42,199/39,672	1.094 (1.080, 1.110)	<0.0001**
	Hypoglycemia	127/126	1.042 (0.815, 1.334)	0.7418	14,959/16,798	0.904 (0.885, 0.924)	<0.0001**
	MASLD	267/202	1.369 (1.140, 1.643)	0.0007**	23,184/19,476	1.227 (1.204, 1.251)	<0.0001**
	OSAS	337/331	1.168 (1.003, 1.359)	0.0448	38,212/33,204	1.274 (1.255, 1.293)	<0.0001**
	Osteoarthritis	165/197	0.854 (0.694, 1.050)	0.1335	19,242/23,856	0.818 (0.803, 0.834)	<0.0001**
	Chronic kidney disease	102/118	0.880 (0.675, 1.147)	0.3438	15,558/20,425	0.759 (0.743, 0.775)	<0.0001**
Psychiatric outcomes	Suicidal ideation	30/78	0.379 (0.249, 0.578)	<0.0001**	1,779/4,963	0.354 (0.335, 0.374)	<0.0001 **
	Alcohol abuse	44/61	0.732 (0.497, 1.079)	0.1136	4,009/7,741	0.516 (0.497, 0.536)	<0.0001**
	Substance use disorder	107/184	0.578 (0.455, 0.733)	<0.0001**	6,572/11,428	0.566 (0.549, 0.584)	<0.0001**
	Depression	299/383	0.779 (0.670, 0.906)	0.0012**	31,309/41,729	0.742 (0.731, 0.753)	<0.0001**
NCID	NCID	291/309	1.024 (0.872, 1.202)	0.7729	28,033/28,107	1.050 (1.033, 1.068)	<0.0001**

HS, hidradenitis suppurativa; GLP-1RA, glucagon-like peptide-1 receptor agonist; HR, hazard ratio; CI, confidence interval; T2D, type 2 diabetes.

** indicates significance after Holm-Šidák correction.

### 3.3 Safety and metabolic profile

Rheumatologic outcomes, including psoriatic arthritis, rheumatoid arthritis, fibromyalgia, collagen diseases, and systemic lupus erythematosus (SLE) showed no significant differences between HS patients treated with GLP-1RA/tirzepatide and controls. These findings were consistent across sensitivity analyses.

Metabolic outcomes indicated higher risk for hyperlipidemia (HR: 1.248, 95% CI 1.096–1.421, *p* = 0.0008), metabolic dysfunction-associated steatotic liver disease (MASLD) (HR: 1.369, 95% CI 1.140–1.643, *p* = 0.0007), and nausea/vomiting (HR: 1.308, 95% CI 1.143–1.497, *p* < 0.0001) in the GLP-1RA/tirzepatide group. No significant differences were observed for constipation, hypoglycemia, osteoarthritis, obstructive sleep apnea syndrome (OSAS), or chronic kidney disease. Findings were consistent in S1, whereas in S2, constipation and OSAS additionally showed an increased risk for the treatment group.

NCID risk was not affected by GLP-1RA or tirzepatide treatment, with consistent results across S1 and S2.

Negative control outcome (presbyopia) showed no significant difference.

In the non-HS benchmark cohort, results were generally consistent, with mixed rheumatologic effects and broadly similar metabolic patterns. Rheumatologic outcomes showed small increases in psoriatic arthritis and fibromyalgia/muscle pain, and a modest reduction in rheumatoid arthritis with rheumatoid factor. Metabolic outcomes showed significant increased risks for hyperlipidemia, MASLD, nausea/vomiting, and OSAS, alongside protective associations for hypoglycemia, osteoarthritis, and chronic kidney disease. NCID showed a small but significant increase in risk. Overall patterns were broadly similar between HS and non-HS cohorts.

### 3.4 HS-associated risk patterns according to GLP-1RA/tirzepatide exposure

To determine whether HS itself was associated with excess comorbidity independent of GLP-1RA/tirzepatide treatment, patients with HS were compared with patients without HS among adults not receiving GLP-1RA/tirzepatide. After correction for multiple comparisons, HS was associated with a significantly higher risk of OSAS (HR: 1.252, 95% CI: 1.085–1.445, *p* = 0.0021) and NCID (HR: 1.262, 95% CI: 1.091–1.460, *p* = 0.0017). HS was also associated with a nominally higher risk of MACE (HR: 1.184, 95% CI: 1.009–1.389, *p* = 0.0383), which did not survive correction for multiple comparisons. No significant differences were observed for other cardiovascular, psychiatric and rheumatologic outcomes (Supplementary Table 2). To assess whether these HS-associated risks persisted under treatment, patients with HS receiving GLP-1RA/tirzepatide were compared with treated patients without HS. HS was associated with higher risk of fibromyalgia/muscle pain (HR: 1.220, 95 % CI: 1.054–1.413, *p* = 0.0075), constipation (HR: 1.207, 95% CI: 1.078–1.350, *p* = 0.0010), nausea/vomiting (HR: 1.138, 95% CI: 1.028–1.261, *p* = 0.0131), OSAS (HR: 1.217, 95% CI: 1.078–1.374, *p* = 0.0015), and NCID (HR: 1.252, 95% CI: 1.101–1.424, *p* = 0.0006). No significant differences were observed for cardiovascular outcomes, other rheumatologic conditions, or psychiatric outcomes (Supplementary Table 3).

## 4 Discussion

This real-world analysis demonstrates that GLP-1RA therapy, including the dual GLP-1/GIP agonist tirzepatide, in patients with HS and comorbid T2D or obesity is associated with significantly reduced all-cause mortality, cardiovascular events, and psychiatric risk, without exacerbating rheumatologic or non-communicable inflammatory outcomes.

While GLP-1RAs are well established for improving glycemic control and promoting weight reduction in patients with T2D and obesity (16), our study extends these observations to HS patients, demonstrating additional cardioprotective effects, including lower risk of MACE, heart failure, myocardial infarction, stroke, ischemic heart disease, and conduction disorders. These findings align with prior TriNetX HS cohorts reporting 18%–27% cardiovascular risk reduction at 5 years of follow-up and are consistent with cardiovascular outcome trials (LEADER, SUSTAIN-6) (13, 17, 18). A recent real-world cohort study in patients with psoriasis and comorbid metabolic disease using a similar design reported comparable reductions in all-cause mortality (HR: 0.219, 95% CI: 0.123–0.391; *p* < 0.001) as well as cardiovascular and psychiatric outcomes under GLP-1RA therapy, supporting consistent systemic benefits of GLP-1RA treatment across distinct chronic inflammatory diseases (15).

These cardiovascular associations were robust across several sensitivity analyses addressing distinct sources of bias. Results were stable when the analysis was repeated at a later data extraction to test temporal reproducibility (S1) and when events within the first three months post-index were excluded to address protopathic bias (S2) (Supplementary Tables 4, 5). To assess potential confounding by adalimumab, we repeated the primary cardiovascular analysis after excluding all adalimumab users (S4). All cardiovascular outcomes remained statistically significant and consistent with the primary analysis (Supplementary Table 7). This is supported by a repeat baseline assessment of the same analytic cohort definition. Due to continuous data accrual within the TriNetX network, this query identified a larger population (cases *n* = 11,647; controls *n* = 7,253) than the original extraction. Within this updated cohort, adalimumab use was reported in 1,018/11,647 (8.7%) of GLP-1RA/tirzepatide patients vs. 463/7,253 (6.4%) of controls, secukinumab in 173/11,647 (1.5%) vs. 59/7,253 (0.8%), and bimekizumab in 10 patients per arm. There was only modest imbalance in adalimumab use between groups (SMD 0.089), below our pre-specified threshold. Adalimumab carries two distinct cardiovascular signals. Heart failure has historically been regarded as a class-related risk of TNF inhibitors, based on a randomized trial of high-dose infliximab in patients with pre-existing advanced heart failure. A recent propensity-matched study in HS reported no association between adalimumab use and incident heart failure, consistent with the present findings (19). By contrast, cohort data in rheumatoid arthritis and psoriasis have associated TNF inhibitors with a reduced risk of MACE, with the magnitude of protection reported to increase with cumulative treatment duration (20). Given this duration-dependence, the observation period in the present study may be insufficient to detect a comparable effect, and a modest, unmeasured influence of adalimumab on MACE risk over longer follow-up cannot be excluded. The stability of S4 nonetheless argues against this exposure as a major driver of the primary cardiovascular associations within the available follow-up. Given the exposure-based cohort definition (≥1-year treatment requirement), we also assessed robustness to immortal time bias using a landmark sensitivity analysis (S5) restricting the risk period to 1–2 years post-index. Associations for MACE, stroke, heart failure, and all-cause mortality were confirmed, indicating these findings are not attributable to immortal time bias alone. Point estimates for myocardial infarction, ischemic heart disease, and conduction disorders remained protective. Their CI overlapped with the primary estimates, but statistical significance was not retained given the shorter accrual window and correspondingly fewer events (Supplementary Table 8). The residual magnitude of the mortality association is addressed further in the Limitations below.

To explore for whom this cardiovascular benefit applies, we performed two further analyses. First, among patients with HS and obesity but without T2D (S3), the association with MACE was not observed (HR: 1.01, 95% CI: 0.58–1.75), and heart failure and ischemic heart disease showed directionally consistent but non-significant trends (Supplementary Table 6). This subgroup captured fewer events than the primary analysis (50 vs. 400 MACE events) despite only a modestly smaller sample size, likely limiting statistical power. The result is hypothesis-generating rather than conclusive. It suggests that diabetes contributes to the cardiovascular benefit seen in the primary analysis. This is consistent with established cardioprotective evidence for GLP-1RAs in diabetic populations (17, 18). Adequately powered studies are needed to test for a cardiovascular effect independent of diabetes status.

Second, treatment-associated reductions in mortality and cardiovascular outcomes were larger in the HS cohort than in the non-HS cohort (Table 2). We tested this directly by comparing treated HS and treated non-HS patients (Supplementary Table 3). No significant differences were observed for cardiovascular outcomes. These findings do not support the hypothesis that patients with HS derive a greater cardiovascular benefit from GLP-1RA/tirzepatide than patients without HS. The larger treatment-associated effects observed in the HS cohort may reflect the higher baseline cardiovascular risk of patients with HS rather than effect modification by HS status.

Beyond the systemic comorbidity outcomes examined in our study, emerging evidence also supports a direct effect of GLP-1RA/tirzepatide on disease activity in HS and related inflammatory conditions. An open-label proof-of-concept study of tirzepatide in 20 adults with moderate-to-severe HS reported that 80% achieved HiSCR at week 24, alongside improvements in quality of life, pain, and anxiety/depression scores (14). This is consistent with recent phase 3b randomized trials in psoriasis and psoriatic arthritis, in which combination therapy with tirzepatide and ixekizumab produced greater improvements in skin clearance, joint disease activity, and weight loss than ixekizumab alone, without new safety signals (21, 22). Together with the present findings, this supports a model in which GLP-1RA/tirzepatide acts on both metabolic and inflammatory pathways relevant to HS, and in which combination with biologic therapy may not only improve disease activity but also reduce the cardiometabolic comorbidity burden that drives excess morbidity and mortality in this population. Mechanistically, this is compatible with reported anti-inflammatory effects of GLP-1RA/tirzepatide on systemic inflammatory markers. HS is characterized by chronic systemic inflammation, with elevated levels of TNF-α, IL-1β and IL-6, that also promote endothelial dysfunction and atherogenesis (23). GLP-1RAs improve glycemic control, accompanied by decreased leptin levels (24). They also suppress NF-*κ*B–mediated inflammation, suggesting that their therapeutic effects may extend beyond metabolic regulation to modulation of inflammatory pathways (25). Randomized, HS-specific trials combining biologic and incretin-based therapy, with endpoints spanning both disease activity and systemic comorbidity, are needed to determine whether this dual approach can prevent, rather than only mitigate, systemic comorbidity.

Our analysis also found that GLP-1RA/tirzepatide use was associated with lower risks of psychiatric outcomes, like suicidal ideation, SUD, and depression. These findings extend meta-analyses showing no increased psychiatric adverse events and potential mental health benefits with GLP-1RAs in T2D and obesity (26). Mechanistically, GLP-1 receptors in brain regions regulating mood, reward, and stress response modulate dopaminergic circuits and neuroinflammation (27, 28). Although underlying mechanisms remain incompletely understood, GLP-1 signaling is expressed in various brain tissues, notably influencing the dopamine reward system and sympathoexcitatory pathways (29, 30). In HS, where chronic pain and inflammation interact via a brain-skin axis, these central effects likely mitigate psychiatric comorbidity (31). Real-world data from patients treated with GLP-1RAs for T2D or obesity further support this, demonstrating reduced risks of suicidal ideation and substance use disorder (32, 33).

GLP-1RAs showed no significant association with rheumatologic outcomes in HS, suggesting a favorable safety profile. This contrasts with mixed effects observed in non-HS adults, where psoriatic arthritis and fibromyalgia/muscle pain were slightly increased, but rheumatoid arthritis risk was reduced. These differences may reflect underlying population characteristics and baseline disease burden. Available GLP-1RA studies in inflammatory skin diseases have mainly examined cutaneous endpoints, with limited rheumatologic data (34).

Metabolic outcomes largely reflected known class effects (35). Hyperlipidemia, MASLD, and nausea/vomiting were increased, whereas constipation, hypoglycemia, osteoarthritis, OSAS, and chronic kidney disease were unaffected in the primary analysis. Gastrointestinal effects may arise from GLP-1RA-induced gastric fundus relaxation and pyloric contraction delaying emptying, activating vagal emetic pathways via central GLP-1 receptors. Hyperlipidemia reflects transient free fatty acid mobilization from rapid visceral fat loss, increasing hepatic VLDL-triglyceride synthesis until metabolic adaptation occurs (4). However, the increases in hyperlipidemia and MASLD may more plausibly reflect differential surveillance and detection bias than biological effects, as GLP-1RA/tirzepatide initiators are likely to undergo more frequent metabolic and hepatic monitoring, whereas less intensive follow-up in comparator groups may contribute to delayed diagnosis and treatment. Their limited magnitude supports tolerability in HS patients with metabolic disease. In adults not receiving GLP-1RA/tirzepatide, HS itself was associated with a higher risk of OSAS, NCID, and a higher MACE risk that did not survive correction for multiple comparisons, underscoring the additional comorbidity burden and the potential of targeted metabolic therapy to mitigate systemic risk.

This study's primary strength lies in the use of a large, multicenter U.S. real-world dataset capturing diverse clinical settings and patient populations. PSM across demographic, clinical, and treatment variables effectively reduced measured confounding and ensured well-balanced cohorts. The inclusion of a broad range of endpoints provides a comprehensive assessment of systemic GLP-1RA/tirzepatide effects in HS. The focus on patients with both HS and metabolic comorbidities reflects a high-risk population in which GLP-1RAs are increasingly prescribed, enhancing direct clinical relevance. Additional strengths include the use of benchmark analysis, Cox proportional hazards models as a confirmatory analytical approach, and inclusion of a negative control outcome. A 2-year follow-up period was selected to ensure sufficient data completeness and adequate cohort sizes and to align with the recent introduction of tirzepatide in 2022, which limits longer-term real-world data availability and supports a uniform observation window across treatment groups (36).

However, the retrospective observational design precludes causal inference. Despite PSM, residual confounding by unmeasured variables such as HS severity or disease duration cannot be excluded, as these parameters are not captured or verifiable within the TriNetX database. Nevertheless, the requirement for systemic HS-directed therapy (including biologic agents, clindamycin, or abscess incision) as an inclusion criterion enriches the cohort for patients with clinically significant disease activity and may partially mitigate this limitation. Treatment adherence was indirectly addressed by requiring a minimum of one year of documented drug exposure. However, this design can introduce immortal time bias. A landmark sensitivity analysis (S5) confirmed that associations for MACE, stroke, heart failure, and all-cause mortality were not attributable to this bias alone, although the mortality association attenuated (HR: 0.240 to 0.399). Because the corrected mortality HR still exceeds effect sizes reported in randomized GLP-1RA cardiovascular outcome trials (e.g., HR: 0.85 in LEADER), residual confounding by indication and healthy-user bias likely remain. Besides this, absolute event counts, particularly in the HS cohort (19 vs. 81 deaths), were modest. This should be considered when interpreting the magnitude of the observed mortality reduction. The cohort was restricted to HS patients with comorbid T2D or obesity receiving systemic pharmacotherapy, resulting in a T2D prevalence (ca. 54% across matched subsets) and insulin use (ca. 26%) exceeding population-based estimates for T2D in unselected HS cohorts (ca. 25%) (37). These findings therefore characterize a metabolically high-risk HS subpopulation already indicated for incretin-based therapy, and should not be extrapolated to HS patients without substantial metabolic comorbidity. Administrative and billing codes may introduce misclassification bias, and generalizability may be limited to U.S. populations. The comparator group also comprised several antidiabetic and anti-obesity drug classes with heterogeneous cardiovascular and metabolic risk profiles. This heterogeneity limits interpretation of the comparator as a single reference standard and may bias especially cardiovascular associations in either direction depending on comparator composition. Drug-class-stratified analyses were not performed due to insufficient power for lower-frequency outcomes within the HS cohort. Future studies with larger sample sizes should compare GLP-1RA/tirzepatide against individual, single-class active comparators.

In conclusion, this real-world study demonstrates that GLP-1RA and tirzepatide use is associated with reduced all-cause mortality, reduced risk of MACE, stroke, and heart failure, and reduced psychiatric morbidity in HS patients with metabolic comorbidities, without increased rheumatologic or inflammatory risk. The results add to previous evidence suggesting potential systemic benefits associated with GLP-1RAs/tirzepatide beyond metabolic control. While the underlying mechanisms require further investigation in prospective HS-specific studies, these findings suggest that GLP-1RAs and dual GLP-1/GIP agonists may warrant further evaluation in the clinical management of metabolically affected HS populations.

## Data Availability

The original contributions presented in the study are included in the article/Supplementary Material, further inquiries can be directed to the corresponding author/s.
