# Supplementary material for: Glucagon-like peptide-1 receptor agonists and tirzepatide are associated with reduced mortality, cardiovascular, and psychiatric risks in patients with hidradenitis suppurativa and type 2 diabetes and/or obesity: a retrospective cohort study
Source: Front Med (Lausanne). 2026 Aug 31;13:1909537. doi: 10.3389/fmed.2026.1909537 (PMC13572214; doi:10.3389/fmed.2026.1909537)
Supplement: Supplementary file 1 [file Supplementaryfile1.pdf]

Supplementary Material

1 Supplementary Figure

Supplementary Figure 1. Forest plot of multivariable Cox proportional hazards models comparing glucagon-like peptide-1 receptor agonist (GLP-1RA) or tirzepatide treatment with other metabolic therapies in patients with hidradenitis suppurativa (HS) and type 2 diabetes (T2D) or obesity. Hazard ratios (HRs) with 95% confidence intervals (CIs) are shown. Models were adjusted for age, sex, race/ethnicity, cardiometabolic comorbidities (obesity, diabetes, hypertension, dyslipidaemia, ischaemic heart disease, chronic kidney disease), lifestyle-related factors (smoking, alcohol-related disorders), concomitant medications (including insulin, cardiac therapies, antidepressants where applicable), and healthcare utilisation. Covariate adjustment varied according to outcome category. HS, hidradenitis suppurativa; T2D, type 2 diabetes; GLP-1RA, glucagon-like peptide-1 receptor agonist; HR, hazard ratio; CI, confidence interval.

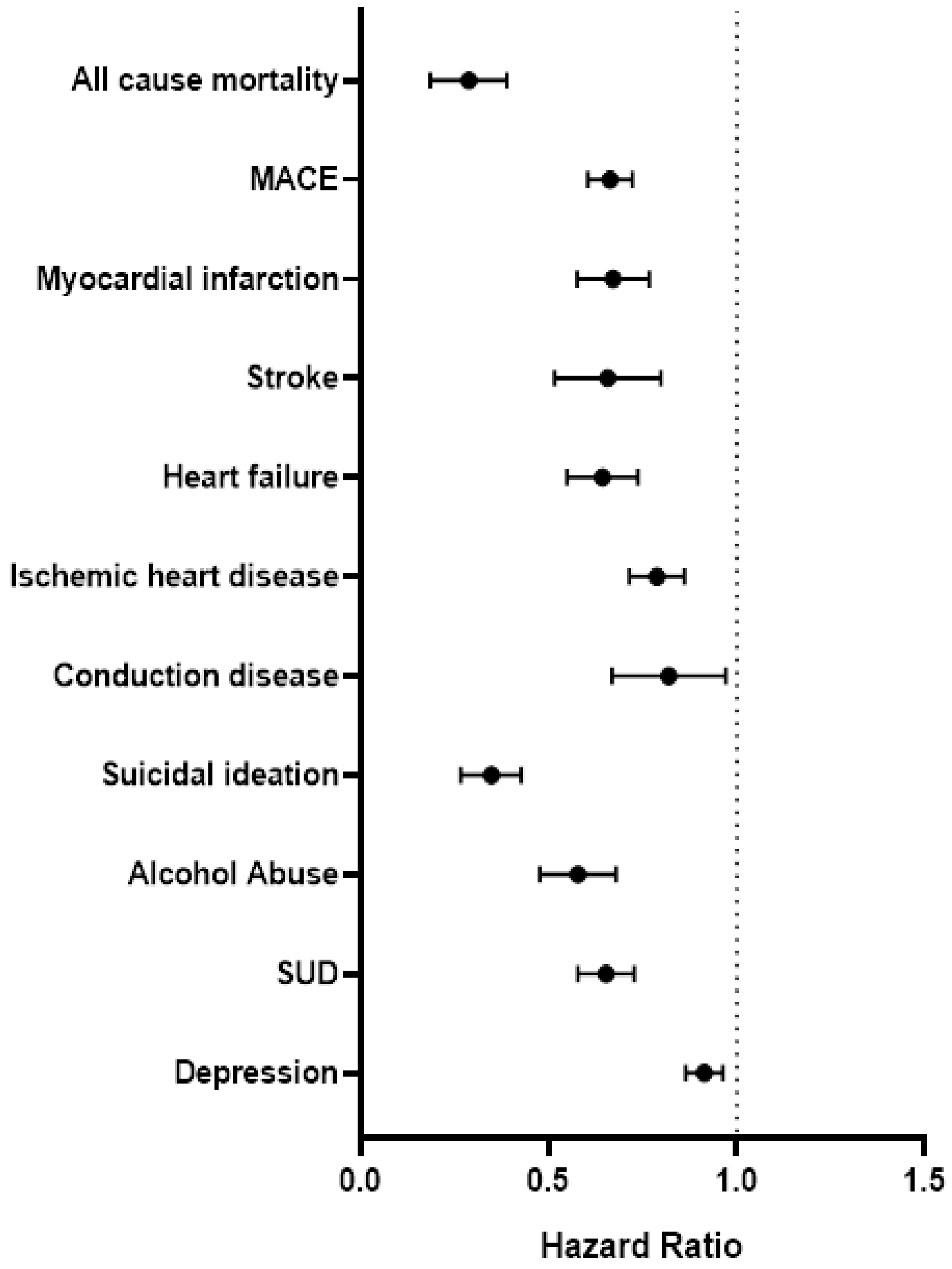

2 Supplementary Tables

Supplementary Table 1: ICD-10-defined outcomes across categories; ICD-10, International Classification of Diseases, Tenth Revision

Category	Outcome	Codes	Description
**Mortality**	All cause mortality	deceased	Death of the patient during the 2-year follow-up period
**Rheumatologic diseases**	Psoriatic arthritis	L40.5	Inflammatory arthritis associated with psoriasis
	Rheumatoid arthritis	M05, M06	Chronic autoimmune inflammatory arthritis
	Fibromyalgia	M79.7, M79.1	Chronic widespread pain syndromes
	Collagen diseases	M35.1, M35	Systemic connective tissue disorders
**Cardiovascular diseases**	MACE	I21, I22, I61, I62, I63, I46, I50, R57.0	Major adverse cardiovascular events
	Myocardial infarction	I21–I23, I25.2	Acute or prior myocardial infarction
	Stroke	I63, I69.3, I60–I62, I69.0–I69.2	Ischemic or hemorrhagic cerebrovascular events
	Heart failure	I50	Clinical heart failure
	Ischemic heart disease	I20, I24, I25.1, I25.3, I25.4, I25.5, I25.8, I25.9, Z95.1	Chronic ischemic coronary artery disease and interventions
	Conduction diseases	I44, I45, I49.5	Cardiac rhythm and conduction abnormalities
**Metabolic diseases**	Hyperlipidemia	E78	Disorders of lipoprotein metabolism
	Constipation	K59.0	Functional constipation
	Nausea/vomiting	R11	Nausea and vomiting
	Hypoglycemia	E11.64, E16.0, E16.1, E16.2	Abnormally low blood glucose levels requiring clinical attention
	MASLD	K76.0	Metabolic dysfunction-associated steatotic liver disease

	Obstructive sleep apnea syndrome (OSAS)	G47.33	Obstructive sleep apnea syndrome characterized by intermittent airflow obstruction during sleep
	Osteoarthritis	M19.9	Degenerative joint disease affecting cartilage and subchondral bone
	Chronic kidney disease	N18.9	Long-standing reduction in kidney function classified by CKD stage
**Psychiatric/ substance use**	Suicidal ideation	T14.91, R45.851	Suicidal thoughts or self-harm-related symptoms
	Alcohol abuse	F10	Mental and behavioural disorders due to harmful or dependent alcohol use
	Substance use disorder (SUD)	F11, F12, F13, F14, F15, F16, F19	Psychoactive substance use disorders
	Depression	F32, F33	Depressive episodes and recurrent depression
**Non-communicable chronic inflammatory diseases**	Autoimmune thyroiditis	E06.3	Chronic autoimmune thyroid inflammation
	Autoinflammatory syndromes	M04.8	Periodic and other autoinflammatory fever syndromes
	Spondyloarthritis	M46.9, M46.1, M45, M02	Inflammatory spondyloarthritides
	Psoriasis	L40	Chronic immune-mediated skin disease
	Psoriatic arthritis	L40.5	Inflammatory arthritis in psoriasis
	Inflammatory bowel disease	K50, K51	Crohn’s disease and ulcerative colitis
	Uveitis	H20	Intraocular inflammatory disease
	Rheumatoid arthritis	M05, M06	Chronic autoimmune inflammatory arthritis

	Systemic lupus erythematosus (SLE)	M32	Systemic autoimmune connective tissue disease
	Fibromyalgia	M79.7, M79.1	Chronic widespread pain syndromes
	Collagen diseases	M35.1, M35.8	Systemic connective tissue disorders
**Negative Controle Outcome**	Presbyopia	H52.4	Age-related loss of near vision not expected to be influenced by treatment

Supplementary Table 2: Disease risk in hidradenitis suppurativa (HS) compared with non-HS controls among patients not treated with GLP-1 receptor agonists (GLP-1RA) or tirzepatide. Hazard ratios (HRs) with 95% confidence intervals (CIs) are shown for rheumatologic, cardiovascular, metabolic, psychiatric and non-communicable inflammatory outcomes (NCID). Analyses were performed in a propensity score–matched (PSM) cohort of adults with obesity or type 2 diabetes (T2D). * indicates statistical significance after Holm–Šidák correction. HS, hidradenitis suppurativa; GLP-1RA, glucagon-like peptide-1 receptor agonist; HR, hazard ratio; CI, confidence interval; NCID, non-communicable inflammatory diseases; PSM, propensity-score matching.

Outcome	Disease	Events (Treatment/Control)	HR (95% CI)	P-value
**Rheumatologic outcomes**	Psoriatic arthritis	—	NA (low event rate)	—
	Rheumatoid arthritis with RF	53/55	0.988 (0.677, 1.440)	0.9487
	Fibromyalgia / muscle pain	281/254	1.142 (0.964, 1.354)	0.1237
	Collagen diseases / MCTD	38/27	1.443 (0.881, 2.363)	0.9018
	Systemic lupus erythematosus	21/13	1.649 (0.826, 3.294)	0.1519
**Cardiovascular outcomes**	MACE	325/279	1.184 (1.009, 1.389)	0.0383
	Myocardial infarction	143/139	1.058 (0.838, 1.336)	0.6366
	Stroke	116/108	1.098 (0.845, 1.427)	0.4847
	Heart failure	244/209	1.192 (0.991, 1.434)	0.0620
	Ischaemic heart disease	267/243	1.126 (0.946, 1.339)	0.1820
	Conduction diseases	168/181	0.951 (0.771, 1.173)	0.6404
**Metabolic outcomes**	Hyperlipidaemia	568/612	0.939 (0.838, 1.053)	0.2800
	Constipation	389/361	1.118 (0.969, 1.290)	0.1270
	Nausea and vomiting	473/473	1.088 (0.958, 1.236)	0.1956
	Hypoglycaemia	157/171	0.948 (0.763, 1.177)	0.6296
	MASLD	240/227	1.089 (0.908, 1.305)	0.3579
	OSAS	401/350	1.252 (1.085, 1.445)	0.0021 **
	Osteoarthritis	236/230	1.096 (0.914, 1.314)	0.3216
	Chronic kidney disease	151/150	1.036 (0.826, 1.298)	0.7619
**Psychiatric outcomes**	Suicidal ideation	92/108	0.866 (0.656, 1.144)	0.3106
	Alcohol abuse	80/103	0.795 (0.594, 1.065)	0.1229
	Substance use disorder	226/201	1.164 (0.963, 1.408)	0.1164
	Depression	488/540	0.979 (0.866, 1.106)	0.7301
**Inflammatory outcomes**	NCID	383/345	1.262 (1.091, 1.460)	0.0017 **

Supplementary Table 3: Disease risk in hidradenitis suppurativa (HS) compared with non-HS controls among patients treated with GLP-1 receptor agonists (GLP-1RA) or tirzepatide. Hazard ratios (HRs) with 95% confidence intervals (CIs) are shown for rheumatologic, cardiovascular, metabolic, psychiatric and non-communicable inflammatory outcomes (NCID). Analyses were performed in a propensity score–matched cohort of adults with obesity or type 2 diabetes (T2D). * indicates statistical significance after Holm–Šidák correction. HS, hidradenitis suppurativa; GLP-1RA, glucagon-like peptide-1 receptor agonist; HR, hazard ratio; CI, confidence interval; NCID, non-communicable inflammatory diseases; T2D, type 2 diabetes.

Outcome	Disease	Events (Treatment/Control)	HR (95% CI)	P-value
**Rheumatologic outcomes**	Psoriatic arthritis	26/19	1.469 (0.813, 2.655)	0.1998
	Rheumatoid arthritis with RF	87/67	1.369 (0.995, 1.882)	0.0526
	Fibromyalgia / muscle pain	384/337	1.220 (1.054, 1.413)	0.0075 **
	Collagen diseases / MCTD	58/59	1.043 (0.726, 1.499)	0.8203
	Systemic lupus erythematosus	18/24	0.796 (0.432, 1.467)	0.4637
**Cardiovascular outcomes**	MACE	289/267	1.151 (0.975, 1.360)	0.0966
	Myocardial infarction	133/130	1.091 (0.856, 1.389)	0.4821
	Stroke	89/100	0.945 (0.710, 1.257)	0.6979
	Heart failure	209/199	1.119 (0.921, 1.359)	0.2568
	Ischaemic heart disease	282/272	1.095 (0.927, 1.293)	0.2869
	Conduction diseases	182/169	1.157 (0.938, 1.427)	0.1717
**Metabolic diseases**	Hyperlipidaemia	659/728	0.957 (0.861, 1.064)	0.4152
	Constipation	644/577	1.207 (1.078, 1.350)	0.0010 **
	Nausea and vomiting	722/743	1.138 (1.028, 1.261)	0.0131 **
	Hypoglycaemia	243/260	1.013 (0.850, 1.206)	0.8886
	MASLD	429/426	1.084 (0.948, 1.239)	0.2398
	OSAS	535/510	1.217 (1.078, 1.374)	0.0015 **
	Osteoarthritis	275/262	1.163 (0.982, 1.378)	0.0792
	Chronic kidney disease	185/175	1.134 (0.922, 1.394)	0.2337
**Psychiatric outcomes**	Suicidal ideation	58/71	0.866 (0.612, 1.226)	0.4178
	Alcohol abuse	84/78	1.150 (0.845, 1.565)	0.3730
	Substance use disorder	195/200	1.068 (0.877, 1.301)	0.5134
	Depression	446/521	0.985 (0.868, 1.118)	0.8207
**Inflammatory outcomes**	NCID	474/455	1.252 (1.101, 1.424)	0.0006 **

Supplementary Table 4: Sensitivity analyses (S1): disease risk in patients with hidradenitis suppurativa (HS), type 2 diabetes (T2D) or obesity treated with GLP-1 receptor agonists (GLP-1RA)/ tirzepatide versus control patients (analysis starting 2 weeks after baseline). Analyses were performed in a propensity score–matched (PSM) cohort of adults with obesity or T2D. * indicates statistical significance after Holm–Šidák correction. HS, hidradenitis suppurativa; T2D, type 2 diabetes; GLP-1RA, glucagon-like peptide-1 receptor agonist; HR, hazard ratio; CI, confidence interval; NCID, non-communicable inflammatory diseases.

Outcome category	Disease	Events (Treatment/Control)	HR (95% CI)	P-value
**All-cause mortality**	All-cause mortality	17/80	0.218 (0.129,0.368)	< 0.0001**
**Rheumatologic outcomes**	Psoriatic arthritis	17/13	1.339 (0.650, 2.758)	0.4261
	Rheumatoid arthritis with RF	52/51	1.025 (0.697, 1.509)	0.8993
	Fibromyalgia / muscle pain	277/289	0.985 (0.836, 1.162)	0.8614
	Collagen diseases / MCTD	40/43	0.953 (0.619, 1.465)	0.8251
	Systemic lupus erythematosus	15/25	0.607 (0.320, 1.152)	0.1229
**Cardiovascular outcomes**	MACE	188/310	0.595 (0.497, 0.714)	<0.0001 **
	Myocardial infarction	79/138	0.577 (0.438, 0.761)	<0.0001 **
	Stroke	59/133	0.529 (0.386, 0.725)	<0.0001 **
	Heart failure	126/234	0.532 (0.428, 0.660)	<0.0001 **
	Ischaemic heart disease	181/251	0.722 (0.596, 0.874)	0.0008 **
	Conduction diseases	113/156	0.741 (0.581, 0.944)	0.0147 **
**Metabolic diseases**	Hyperlipidaemia	511/435	1.236 (1.088, 1.405)	0.0011 **
	Constipation	380/339	1.141 (0.986, 1.321)	0.0772
	Nausea and vomiting	486/411	1.314 (1.152, 1.498)	<0.0001 **
	Hypoglycaemia	127/128	1.034 (0.809, 1.322)	0.7895
	MASLD	258/209	1.294 (1.078, 1.553)	0.0055 **
	OSAS	336/337	1.155 (0.993, 1.343)	0.0615
	Osteoarthritis	159/192	0.844 (0.684, 1.042)	0.1136
	Chronic kidney disease	107/127	0.857 (0.663, 1.109)	0.2400
**Psychiatric outcomes**	Suicidal ideation	32/88	0.359 (0.240, 0.539)	<0.0001 **
	Alcohol abuse	47/72	0.637 (0.439, 0.924)	0.0166 **
	Substance use disorder	125/205	0.602 (0.482, 0.752)	<0.0001 **
	Depression	320/427	0.752 (0.651, 0.870)	0.0001 **
**Inflammatory outcomes**	NCID	287/312	1.009 (0.859, 1.184)	0.9141

Supplementary Table 5. Sensitivity analyses (S2): disease risk in patients with hidradenitis suppurativa (HS), type 2 diabetes (T2D) or obesity treated with GLP-1 receptor agonists (GLP-1RA)/ tirzepatide versus control patients (excluding events within the first 3 months post-index date.) Analyses were performed in a propensity score–matched (PSM) cohort of adults with obesity orT2D. * indicates statistical significance after Holm–Šidák correction. HS, hidradenitis suppurativa; T2D, type 2 diabetes; GLP-1RA, glucagon-like peptide-1 receptor agonist; HR, hazard ratio; CI, confidence interval; NCID, non-communicable inflammatory diseases.

Outcome	Disease	Events (Treatment/Control)	HR (95% CI)	P-value
**All-cause mortality**	All-cause mortality	22/87	0.261 (0.164, 0.417)	<0.0001 **
**Rheumatologic outcomes**	Psoriatic arthritis	16/11	1.505 (0.699, 3.245)	0.2930
	Rheumatoid arthritis with RF	48/43	1.131 (0.749, 1.706)	0.5586
	Fibromyalgia / muscle pain	235/245	0.988 (0.826, 1.182)	0.8934
	Collagen diseases / MCTD	26/40	0.671 (0.409, 1.099)	0.1108
	Systemic lupus erythematosus	—	NA (low event rate)	—
**Cardiovascular outcomes**	MACE	164/238	0.683 (0.560, 0.834)	0.0002 **
	Myocardial infarction	70/115	0.617 (0.458, 0.831)	0.0013 **
	Stroke	49/89	0.563 (0.397, 0.798)	0.0011 **
	Heart failure	109/188	0.576 (0.455, 0.730)	<0.0001 **
	Ischemic heart disease	156/204	0.770 (0.625, 0.948)	0.0136 **
	Conduction diseases	93/139	0.685 (0.527, 0.891)	0.0046 **
**Metabolic diseases**	Hyperlipidemia	454/355	1.354 (1.178, 1.555)	<0.0001 **
	Constipation	378/317	1.225 (1.055, 1.422)	0.0077 **
	Nausea and vomiting	424/385	1.222 (1.065, 1.403)	0.0043 **
	Hypoglycemia	124/126	1.037 (0.809, 1.329)	0.7726
	MASLD	246/200	1.298 (1.077, 1.565)	0.0060 **
	OSAS	323/298	1.263 (1.079, 1.479)	0.0036 **
	Osteoarthritis	147/190	0.795 (0.641, 0.986)	0.0365
	Chronic kidney disease	96/116	0.856 (0.653, 1.122)	0.2595
**Psychiatric outcomes**	Suicidal ideation	32/82	0.389 (0.259, 0.586)	<0.0001 **
	Alcohol abuse	40/57	0.719 (0.480, 1.078)	0.1444
	Substance use disorder	108/174	0.614 (0.483, 0.780)	<0.0001 **
	Depression	277/352	0.803 (0.686, 0.940)	0.0062 **
**Inflammatory outcomes**	NCID	271/314	0.960 (0.816, 1.129)	0.3525

Supplementary Table 6. Sensitivity analyses (S3): disease risk in patients with hidradenitis suppurativa (HS) and obesity without type 2 diabetes (T2D) treated with glucagon-like peptide-1 receptor agonists (GLP-1RA)/tirzepatide versus control patients. Analyses were performed in a propensity score–matched (PSM) cohort of adults with HS and obesity after exclusion of patients with T2D. HS, hidradenitis suppurativa; T2D, type 2 diabetes; GLP-1RA, glucagon-like peptide-1 receptor agonist; HR, hazard ratio; CI, confidence interval.

Outcome	Disease	Events (Treatment/Control)	HR (95% CI)	P-value
All-cause mortality	All-cause mortality	—	NA (insufficient event rate)	—
Cardiovascular outcomes	MACE	25/25	1.006 (0.577–1.751)	0.9843
	Myocardial infarction	—	NA (insufficient event rate)	—
	Stroke	—	NA (insufficient event rate)	—
	Heart failure	14/16	0.879 (0.429–1.802)	0.7250
	Ischemic heart disease	21/30	0.706 (0.404–1.233)	0.2189
	Conduction diseases	23/28	0.843 (0.485–1.463)	0.5427

Supplementary Table 7: Sensitivity analyses (S4): disease risk in patients with hidradenitis suppurativa (HS) and obesity or type 2 diabetes (T2D) treated with glucagon-like peptide-1 receptor agonists (GLP-1RA)/tirzepatide versus control patients, after exclusion of adalimumab users. Analyses were performed in a propensity score–matched (PSM) cohort after exclusion of patients receiving adalimumab. * indicates statistical significance after Holm–Šidák correction. HS, hidradenitis suppurativa; GLP-1RA, glucagon-like peptide-1 receptor agonist; HR, hazard ratio; CI, confidence interval.

Outcome	Disease	Events (Treatment/Control)	HR (95% CI)	P-value
Cardiovascular outcomes	MACE	176/289	0.592 (0.491–0.715)	<0.0001**
	Myocardial infarction	85/140	0.608 (0.464–0.796)	0.0002**
	Stroke	60/115	0.522 (0.382–0.713)	<0.0001**
	Heart failure	124/211	0.575 (0.461–0.718)	<0.0001**
	Ischemic heart disease	179/235	0.761 (0.626–0.924)	0.0057**
	Conduction diseases	107/149	0.722 (0.563–0.926)	0.0099**

Supplementary Table 8: Sensitivity analyses (S5): disease risk in patients with hidradenitis suppurativa (HS), type 2 diabetes (T2D) or obesity treated with GLP-1 receptor agonists (GLP-1RA)/ tirzepatide versus control patients after landmark analysis with outcome ascertainment starting 1 year after the index date. Analyses were performed in the propensity score–matched (PSM) cohort. * indicates statistical significance after Holm–Šidák correction. HS, hidradenitis suppurativa; GLP-1RA, glucagon-like peptide-1 receptor agonist; HR, hazard ratio; CI, confidence interval.

Outcome	Disease	Events (Treatment/Control)	HR (95% CI)	P-value
All-cause mortality	All-cause mortality	20/52	0.399 (0.238–0.669)	0.0003**
Cardiovascular outcomes	MACE	97/151	0.633 (0.490–0.817)	0.0004**
	Myocardial infarction	50/63	0.815 (0.562–1.182)	0.2801
	Stroke	31/62	0.512 (0.332–0.787)	0.0019**
	Heart failure	61/112	0.541 (0.396–0.740)	<0.0001**
	Ischemic heart disease	89/104	0.872 (0.657–1.158)	0.3438
	Conduction diseases	64/74	0.894 (0.640–1.250)	0.5125
